# The Interacting Axes of Environmental, Health, and Social Justice Cumulative Impacts: A Case Study of the Blueberry River First Nations

**DOI:** 10.3390/healthcare4040078

**Published:** 2016-10-18

**Authors:** Maya K Gislason, Holly K Andersen

**Affiliations:** 1 Health Sciences, Simon Fraser University, Burnaby, BC V5A 1S6, Canada; 2 Philosophy Department, Simon Fraser University, Burnaby, BC V5A 1S6, Canada; handerse@sfu.ca

**Keywords:** cumulative impacts, health, indigenous, equity, ethics, intensive resource extraction, environment, justice, mechanisms, community

## Abstract

We consider the case of intensive resource extractive projects in the Blueberry River First Nations in Northern British Columbia, Canada, as a case study. Drawing on the parallels between concepts of cumulative environmental and cumulative health impacts, we highlight three axes along which to gauge the effects of intensive extraction projects. These are environmental, health, and social justice axes. Using an intersectional analysis highlights the way in which using individual indicators to measure impact, rather than considering cumulative effects, hides the full extent by which the affected First Nations communities are impacted by intensive extraction projects. We use the case study to contemplate several mechanisms at the intersection of these axes whereby the negative effects of each not only add but also amplify through their interactions. For example, direct impact along the environmental axis indirectly amplifies other health and social justice impacts separately from the direct impacts on those axes. We conclude there is significant work still to be done to use cumulative indicators to study the impacts of extractive industry projects—like liquefied natural gas—on peoples, environments, and health.

## 1. Introduction

The purpose of this discussion piece is to reflect on the environmental, health, and ethical dimensions of the impacts of intensive natural resource extraction (IRE) on Northern British Columbia (BC) in Canada. Within the province of BC, northern BC comprises over 70% of the landmass of the province, less than 10% of the total population, over 50% of the Indigenous population and is the site of approximately 65% of all the active or proposed projects related to intensive resource extraction (IRE) and manufacturing [1]. In this region, whether from mining, fishing, forestry, or oil and gas, communities are directly and indirectly impacted by intensive resource extraction (CIIREs). The Northern Health Authority, responsible for the public health of this region, describes that resource projects can have not only positive impacts such as economic growth but also negative impacts on health as it puts pressure on health care infrastructure and services, places strains on communities, intensifies the impacts of the socio-economic determinants of health and increases environmental health impacts [2]. This region is, therefore, a clear illustration of the ways in which human health and wellbeing is shaped not only by the social and economic contexts and conditions within which people are “born, live, learn, love, work, play, worship, and age” [3] but also by ecological systems and services. In other words, in Northern BC we can clearly see that people’s health not only begins in their “homes, schools, workplaces, neighborhoods, and communities” [4] (p. B-6) but also in the air, water, forests, and soils used to sustain and nourish our health and enable and enrich our social, cultural, and economic lives [5].

Amidst urgent efforts to gather robust evidence about the impacts of intensive resource extraction in order to guide public health and health care delivery as well as to better govern the sustainable development of communities, the ethical dimensions of the impacts of IRE are not often adequately contemplated and the insights gained from these exercises properly reflected in formal processes. While it is recognized that public health has close connections to human rights [6,7,8], these are often considered in terms of health as a human right. We highlight additional mechanisms by which rights and health are connected, in terms of the meaningful exercise of Treaty rights for First Nations in Canada, mediated through environmental health in treaty-protected reserve areas.

This paper considers the impacts of IRE on Northern BC in terms of the ethical ramifications of cumulative impacts on vulnerable groups of people in this area. Cumulative impacts, are “changes to the biophysical, social, economic, and cultural environments caused by the combination of past, present and ‘reasonably foreseeable’ future actions. Cumulative impacts can be positive or negative” [9]. The methodologies by which environmental impacts on the one hand, and health impacts on another, are measured themselves contain moral commitments about the value of single indicators versus measures of cumulative impacts. Further, the term “health” has a dual role to play, as it is widely used in environmental ethics as a way of measuring ecological integrity and resilience for example, and used in analogous ways to measure health and wellbeing in humans. Currently, urgent efforts are being made by health authorities and scholars to gather robust evidence on human health, including efforts to better understand how environmental and animal health are related, in order to better guide public health and health care delivery responses. Central to this work is the assembling of robust evidence intended to inform the better governance of sustainable community development initiatives. This paper is particularly interested in the ethical dimensions of the methodologies used for measuring the impacts of IRE—including within formal environmental, health, and risk assessment processes—and the ethical concerns that arise from current practices.

To illustrate how the links between health and rights interact to impact social and environmental landscapes, and how people living in communities in Northern BC embody these multiple factors, we offer a case study which focuses on extractive industry projects in the treaty-protected area of the Blueberry River First Nations (BRFN). Importantly, this case exhibits features that allow for generalization to other related cases in an illuminating way. We draw on discussions in the philosophy of science literature regarding mechanisms and methodologies of measuring environmental impacts in order to draw parallels between environmental, health, and social justice impact measurements while highlighting the pathways by which direct harm can be amplified in indirect ways through harms to other parameters. We also applaud advances that have been made in measuring harm to the environment which extends from strengthening individual indicators through to the commitment to develop models where the cumulative impact of multiple factors are measured and not merely reduced to a few key indicators. A parallel movement for cumulative impact on health is still in its nascent stages. Equally important is the development of research and governance which acknowledges the complex ways in which human health is inextricably linked to animal health and ecological integrity.

While methodological innovation occurs, however, and governmental commitments are made, for example by the BC government, much progress has not been made and the social justice impacts associated with these projects accrue, sometimes in unexpected ways. We will discuss, for example, “engagement fatigue” as an impact on communities and community members that derives from the manner in which First Nations are consulted about the other kinds of impacts of these projects. Our case analysis of the pressures facing the BRFN highlights several key conceptual distinctions that draw out the ethical implications of various methodologies for assessing the potential, extent, and character of impacts of proposed extraction projects.

## 2. Materials and Methods

Literature reviews on the subjects of the public health impacts of intensive resource extraction on health and on treaty rights of First Nations groups in the area were conducted and the findings presented in the third Results Section. Both research articles and policy documents were consulted in this analysis. We rely on a more traditional philosophical analysis to highlight key items for discussion and to suggest ways to frame this issue that can be used to guide further investigations, which is found in the fourth Discussion and fifth Conclusion Sections. Our framework for philosophical analysis is drawn from contemporary discussion in Philosophy of Science, including discussions of mechanisms as a form of scientific explanation [10,11,12,13], discussions of philosophy of ecosystems [14,15,16], and discussions of values in science [17,18]. This approach is easily applied to environmental issues such as ecosystem harm, and to the ways in which differences between different indicator methodologies are value-laden in their effects, since it was developed to be applied to exactly those kinds of cases. We extend this approach by analyzing health and social justice indicators in terms of mechanisms that are analogous in relevant ways to the environmental ones. This utilizes a familiar concept of intersectionality [19] but implements it in a new approach by applying it to axes along which to measure harm, with identifiable biological and social mechanisms by which these axes interact at their intersections. 

## 3. Results

This section provides more detailed information on the case study.

We propose three key axes along which to identify types of potential harm that can vary in severity. These three axes are: Environment; Health; and Social Justice. Part of our framing of the results in this way is meant to highlight ethical considerations pertaining to the methodologies used to measure impacts along each axis.

### 3.1. Blueberry River First Nations

**Figure 1:**
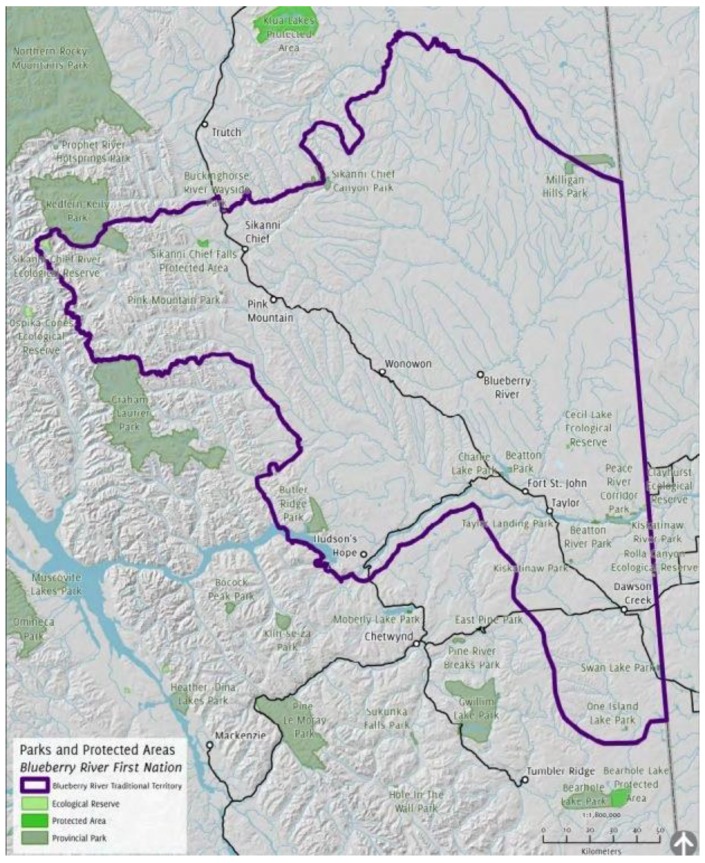
Parks and protected areas: Blueberry rivers first nations (from the 2017 Atlas of cumulative land disturbance).

The Blueberry River First Nation’s (BRFN) peoples are Dane-zaa (Beaver) and are part of the Northern Athapaskan language group, although this nation now includes both Dane-zaa and Cree speakers [20] (p. 3). Today, the primary BRFN Indian Reserve (I.R. No. 205) is located at Blueberry River, approximately 80 km northwest of Fort St. John, BC (see Figure 1 [21]). A second BRFN Indian Reserve is located at the southern half of Beaton River (I.R. No. 204). These two reserves are also nested within other jurisdictional and geographical boundaries including the Peace Region in BC and Treaty No. 8, established in 1899 [22] (p. 39). The Treaty 8 area is comprised of 324,900 sq. miles of territory which spans “the northern half of Alberta, the northeast quarter of British Columbia, the northwest corner of Saskatchewan, and the area south of Hay River and Great Slave Lake in the Northwest Territories” [23]. The Peace River region has long been acknowledged to contain a wealth of natural resources. Scholars observe that, in the late 19th century, Treaty 8 was demarcated “partially in response to the growing tensions between Aboriginal peoples and settlers over land and resources [24] (p. 138). Against a history of colonization, pulses of pressure on this area have also occurred; for example, particular requests by the government were made to the Dane-zaa to relinquish further territory and rights to natural resources after both the First and Second World War [25] (p. 11), [26] (p. 2), [27] (p. 15). Today, the impacts of a history of intensive resource extraction are increasingly visible as this single land base has undergone widespread change as it is here that a number of industrial interests converge.

### 3.2. Environmental Impacts

In order for a large scale project to be executed, such as a mine, pipeline, or dam, approval is required, which includes undertaking an Environmental Assessment (EA) intended to function as a cooperative endeavor between provincial and federal processes. With any large scale development in Canada, the proponent is also required to conduct consultations with the general public as well as with impacted Aboriginal groups (referred to as “Project Area Aboriginal Groups”). Projects are approved on a case by case basis, once they have met the required conditions. The impact of each project is thus considered separately from that of other projects in the area, even if they will have overlapping impacts on the same communities, geographical areas, and ecosystems. Cumulative impact of the totality of projects, either those currently active or those active plus those that have already been completed, is not taken into account as part of the EA.

**Figure 2:**
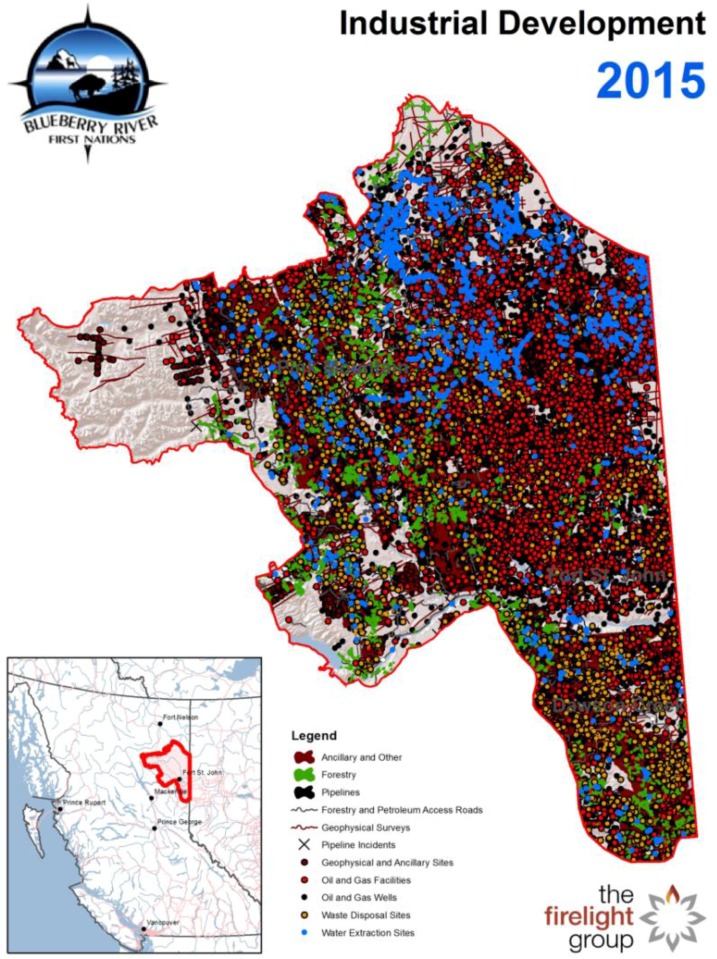
This map shows the cumulative impacts of industrial development activities in 2015 in the Blueberry River First Nations traditional territory [35].

This disaggregated approach stands in stark contrast to the realities of intensive resource development occurring in Canada, including in Northern BC. Scholars following these developments describe that the majority of the “mature forests, rivers, wetlands, and other ecosystems have already been changed by logging, mining, oil and gas development (conventional and non-conventional), water withdrawals and stream crossings, large-scale hydro development (e.g., W.A.C. Bennett Dam), agricultural conversion and other industrial developments” [28] (p. 24). Others note that this region harbours substantial sources of resources and potential energy generation which will continue to attract industrial interest. Recent developments include, “2 large-scale hydroelectric dams, 11 mines (gold-copper, coal), 8000 oil and gas well sites, 8 wind farms, various support facilities, 10,000 pipelines, numerous power lines, and smaller uses such agriculture and guide-outfitting” [29] (p. 7). The BRFN reserve is notably impacted by such this range of industrial activity and the federal and provincial ambitions that enable them [28] (p. 23). For example, “more than one-half of all the oil and gas facilities are within the Beatton watershed” [30] (p. 25) and in this northeastern part of the territory, 90.8% of the territory has been disturbed as have the community, which has already been evacuated due to real or potential gas leaks in the pipelines [28] (p. 24). Chief Yahey shares: “Blueberry’s ancestors would not recognize our territory today. It is covered by oil and gas wells, roads, pipelines, mines, clear cuts, hydro and seismic lines, private land holdings, and waste disposal sites, amongst other things…The pace and scale of development have accelerated in the last 25 years, and are now at unprecedented levels.” [31]. While each sector is influenced by industrial and economic push and pull factors, the recent bust in oil prices has overall dampened activity in the region. Nevertheless, the impacts of exploration have already been tremendous as has been the development of supporting infrastructure. For example, while there are currently 19 proposals for LNG terminals along B.C.’s coast and only a few terminals will actually reach completion, their creation alone places considerable pressure to increase the development of B.C.’s shale gas resources in the northeast—one of many forms of IRE in the region. In addition, for communities farther up the supply chain, the loss of revenue have also been significant. Offering frameworks for contextualizing and integrating the impacts of liquid natural gas, for example, the Cumulative Impacts Research Consortia (CIRC) proposes that one way to understand the impact on communities is to consider how they are distributed across the supply chain using the schema of “upstream” gas producing regions (e.g., Northern Rockies), “midstream” transportation areas (e.g., areas with pipelines including the Skeena and Bulkley-Nechako), and “downstream” processing and export communities (e.g., the North Coast) [32]. The figure below compares industrial activity in the BRFN territory as of 2015 [33] (see Figure 2). While each project represented as a dot on the map has been approved separately, for the communities impacted, the impacts accrue. The cumulative impacts, the community alleges, have had adverse impacts to the “land, water, fish and wildlife” in their territory [34] (p. 7) which has resulted in the loss, displacement and the curtailment of their ability to exercise treaty rights [34] (p. 8).

The rate and scale of industrial development has accelerated over time, with implications for how effectively humans, animals, and ecosystems will be able to adapt to the changes occurring. The BRFN Land Stewardship Framework documents some of these changes and underscores that the BRFN territory has one of the highest burdens of IR in all of BC [35]. For example, in 1950 less than 50% of the Blueberry watershed was disturbed yet by 2016, 84% of the whole territory is within 500 m of industrial disturbance [36] (p. 6).

The BRFN and the David Suzuki Foundation commissioned Ecotrust Canada to produce, based on government data and existing maps, the “Atlas of Cumulative Landscape Disturbance in the Traditional Territory of Blueberry River First Nations” [37]. This report finds that, to date, within BRFN territory, the BC government has authorized the construction of more than “2600 oil and gas wells, 1884 km of petroleum access and permanent roads, 740 km of petroleum development roads, 1500 km of new pipelines and 9400 km of seismic lines and approximately 290 forestry cutblocks were harvested” [37] (p. 6). David Suzuki, describes that all of this development one encounters road, seismic line, or industrial infrastructure within every half a kilometer and emphasizes that: “A natural functioning landscape with species including large predators requires a maximum density limit of 0.6 km of linear disturbances—roads and seismic and transmission lines—per square kilometer. Blueberry River has 2.88 km of linear disturbance per square km, totaling 110,300 km—including 45,603 km of seismic lines constructed over the past 10 years, nearly eight times the length of the Trans-Canada Highway from Vancouver to Halifax” [38].

The impacts of IRE extraction are of concern at present but will also have an effect on future generations—a fact which underscores the moral imperative of addressing these issues with focus and a sense of urgency, particularly in light of the possibility that thresholds and tipping points in the region may have already been reached.

One of the many projects that is having an impact is BC Hydro’s Site C Clean Energy Project (Site C) [39,40] which is proposed to be an earthfill dam and hydroelectric generating station on the Peace River in northeast B.C. The plan is for the dam to be 1050 m long and 60 m high above the riverbed while the Site-C reservoir is intended to stretch 83 km long and extend the current width of the river by 2–3 times. This will require the flooding of 5430 hectares of land [27] (p. 4). While the project is marketed as a “clean energy project” a study on the role of the Peace River Region in mitigating against climate change shows that the construction of Site C will not only disrupt the ability of the region to sequester Green House Gases (GHG) in the valley’s plants and soils but will also produce significant GHG emissions in its construction, which is planned to span approximately 10 years [39].

BC Hydro, in its efforts to develop the proposed Site C Dam, reports it has conducted over 500 consultations. It also initiated an EA which is intended to produce an evidence-based understanding of the baseline conditions and background information about the potential impacts of the project and to aid in planning to address their effects. Following the protocols laid out for Environmental Assessment (EA) in BC, 22 valued components (VC) were identified for the EA which fall under five “pillars”: (1) environmental, (2) economic, (3) social, (4) heritage, and (5) health [37] (p. 24). BC Hydro confirms that the VC assessment of potential effects was conducted by identifying which pillars that would be effected by the proposed project and then to ascertain which are the technically and economically feasible mitigation strategies. The environmental assessment certificate (EAC) was eventually approved subject to 77 conditions. However, for the BRFN, the building of this dam would represent the potential loss of “5000 hectares of productive land base” which would “lead to the effective loss of rights to hunt elk, moose, caribou, and other animals; loss of bear habitat; mercury contamination of fish; downstream effects; and cumulative effects from the Project in combination with extensive existing and future developments, particularly oil and gas development in their territory...” [37] (p. 424). With approximately 73% of the industrial disturbances occurring with 250−500 m of BRFN traditional territory, the impacts on human, animal, and ecological health are cumulative and require urgent response.

### 3.3. Health Impacts

Current work is explicitly exploring the key areas of impact on human health which include physiological, psychological, cultural/spiritual, and ecological domains [41,42]. Agencies responsible for the public health and wellbeing of people in northern BC, the Northern Health Authority (NHA), and the First Nations Health Authority (FNHA), are reporting increased public health service needs in boom times where there are intensive resource extraction initiatives and large temporary work camps. Health challenges also emerge during bust eras when the sector goes into decline and communities are left in economic and social turmoil, housing and food instability increases, mental health issues can lead to higher rates of substance misuse as well as domestic and sexual violence and related infection and trauma sequela [43]. At the same time that the health authorities are trying to assemble a picture of the precise health needs of both Indigenous and non-Indigenous communities in order to respond to the increasing rate and scale of emergent requests, research attention is being placed on understanding the mechanisms through which public health is and may be impacted by intensive resource extraction writ large as well as by boom and bust cycles more specifically. For the BRFN, however, understanding the health impacts to the community requires considering not only the environmental, social, and cultural health impacts generally but also the three cornerstone impacts expressed within Indigenous Determinants of Health scholarship, which are the impacts of IRE on the community’s: 1. Connection to land; 2. Cultural continuity; and 3. Ongoing impacts of colonization [44]. It also underscores the importance of mainstream health services responding in culturally sensitive and regionally relevant ways to existing and emergent health issues.

### 3.4. Social Justice Impacts

The BRFN, despite all this development, continue to be not only culturally and spiritually connected to the Peace River but also reliant upon the land [37] (p. 102). One thing that has remained constant over the centuries is that country food, including wildlife, and traditional practices remain an important part of life and survival for BRFN. For example, the BRFN hunt both sides of the Peace River for moose, elk, deer, bear, mountain sheep, and caribou [27]. They also fish for a range of species including dolly varden, rainbow trout, bull trout, kokanee, jackfish/pike, pickerel/walleye, suckers, whitefish, lingcod, and grayling [25] (p. 5). For modern Indigenous peoples, “the land remains an important resource for subsistence purposes, as well as the foundation of their way of life, culture, and identity” [10] (p. 3). Olson and DeRoy underscore that their research reinforces the message that “the ability of BRFN members to practice their treaty rights has been significantly impacted by industrial development (forestry, hydro, mining, oil and gas, and the corresponding influx of settlers) in and around BRFN territory [10] (p. 4). They go on to illustrate that, in order to exercise their treaty rights, for example to “hunt, trap, fish, and practice their traditional mode of life and culture”, the BRFN must live within intact ecosystems [10] (p. 4). Yet, Olson and DeRoy, go on to argue, development initiatives have led to impacts which include “environmental degradation, contamination of the land, air and water, habitat fragmentation, and disturbance of key sites to BRFN (i.e., calving grounds, areas for harvesting medicinal plants etc.), amongst others” [10] (p. 4). For example, if BC Hydro’s Site C dam goes forward, loosing “5000 hectares of productive land base” which would “lead to the effective loss of rights to hunt elk, moose, caribou, and other animals; loss of bear habitat; mercury contamination of fish; downstream effects; and cumulative effects from the Project in combination with extensive existing and future developments, particularly oil and gas development in their territory...” [37] (p. 424). The inextricable link between animal and environmental health and its core importance to BRFN health underscore that to accurately understand cumulative impacts, the variables considered must reflect the realities and domains of relevance for the people and places that are impacted.

For the BRFN, the approach to IRE undertaken by private companies and enabled by federal and provincial governance bodies, led them to file a civil claim against the Province of BC on 3 March 2015 for breaching their fiduciary obligations [34,45]. The first statement of facts in the claim requests that the Province: “stop the consistent and increasingly accelerated degradation of the Nation’s traditional territory, and to protect and enforce the Nation’s constitutionally protected rights against the cumulative impacts of Crown authorized activities on their traditional territory” [34] (P. 2). The BRFN alleged that the cumulative effect of industrial development in the Treaty 8 area has made or will soon make it impossible for its members to meaningfully exercise treaty rights such as hunting and fishing and therefore argue that the Crown has breached treaty obligations, as well as interim and permanent injunctions to prohibit the Province from doing or permitting any activities that amount to a further breach” [46] (p. 1). At the center of this lawsuit is the argument that “the cumulative effect of the industrial development in the Treaty 8 area had become so extensive that it amounted to a violation of a treaty right” [46] (p. 2). They maintain, “each new incursion becomes more significant than the last. In that sense, any portion of the overall loss in this case, if it is found to exist, should be characterized as irreparable harm” [46] (p. 2). Court challenges like this by First Nations are made in a context of neoliberal visions to economic growth and clear and consistent track records of a disregard for the social and ecological sustainability in the region. Financial incentives, however, can impact the priorities and practices not only of industries but also can influence how governmental and nongovernmental bodies alike interact with First Nations. For example, BC Hydro recently offered land transfers of more than 5000 hectares to eight First Nations bands; however, there is secrecy around what deal each band has been offered and concern that these arrangements would not only serve to divide First Nations communities but also reduce public access to crown land in the Peace River region [47,48]. At present, there has been a great deal of criticism about BC Hydro’s strategy.

All of this is occurring in a region dominated by IRE where communities, sometimes reliant on single resource economies, are significantly impacted by national and international economics. Boom and bust economies, whether analyzed through the lens of labor studies or public health, are understood to bring with them myriad problems which significantly affect the most vulnerable, as will be described in the following section. Booms in resource economies are characteristically unstable and typically lead to eventual economic busts and in June 2015, the LNG industry in northern BC entered a bust cycle. In addition to the decline in oil prices and a reduced demand for LNG globally, Canadian LNG can be hard to sell competitively on the international market as it is costly to produce. It is an unenviable task to weather boom and bust cycles. For people living in communities in northern BC, this means being buffeted about by fluctuations in economies over which they have little or no control, it often means that temporary workers leave and those who are long-term residents of host communities are left to find ways to live within towns that are being dismantled socially and economically. For all residents of the region, but most significantly for Indigenous peoples, it also means that the local populations are left to resource essential ecosystem services—such as clean air, water, soil, country foods, and wildlife—from landscapes which have been dramatically altered and often contaminated.

## 4. Discussion

With these details in place, we turn to a more philosophical analysis of the three axes we proposed by which to organize the effects of IRE: environmental, health, and social justice. Drawing from this case study background, we consider here how these axes intersect, and highlight mechanisms by which the impact of one axis can be amplified through its interaction with another intersecting axis. The mechanisms discussed here are not exhaustive of the ways in which IRE may impact, but are conceptually illustrative of how the intersections of these already identified axes can serve as the locus to amplify negative impacts from one axis to indirectly perpetuate further negative impacts on the other axes. We also highlight the ethical dimension of choosing to measure harm in terms of individual indicators and individual projects, rather than in terms of cumulative impacts. This use of individual indicators involves a commitment to reductionism that Morrison has argued is an inappropriate methodology for biological systems, including ecosystems [49]. We extend her intersectional analysis to the health and social justice axes.

There are ethical implications for practices which use discrete and disaggregated indicators to measure impact, which raises interesting questions about current practices when analyzed through the lens of environmental ethics. A key distinction is that of measuring impact by a collection of indicators, each of which are assessed separately in terms of a threshold, for instance; versus measuring impact cumulatively across a range of indicators where the overall impact of the indicators is considered in addition to whether any individual indicator crosses a threshold. In terms of our case study, along each of the axes, specific indicators are used to operationalize what can and will be measured in terms of the impact of various IRE projects. The indicators thus serve to stand in for the axis as a whole, such that the way in which these indicators are chosen, how they are measured, and how impacts on them are assessed, comprised the methodology used to maintain the integrity of each axis. A commitment to using measures that reflect cumulative harm is thus a commitment to more accurately measure the impact not merely of one project, but of the entirety of the projects.

This is a commitment with ethical dimensions, since a full-fledged commitment to prevent or mitigate harm requires consideration of cumulative as well as individual impacts. By dividing the measurement of impacts into single-project EAs, and considering each project in isolation from others in the area, the overall EA and consultation process is in principle unable to reflect the full picture of how IRE is affecting the local health of people, animals, and the environment. There is no further point in any given process for approval in current British Columbia practice such that the full extent of all projects up to that point can be considered. By using individual indicators and/or by failing to have a separate process whereby the cumulative impacts of multiple projects across multiple potential avenues of harm, there is a structural failure of the process for communities like the BRFN. The choice of methodology for measuring harm itself covers over the full extent of the harm, while at the same time appearing to have the appropriate scientific credibility.

The ethical implications of measuring each of these three axes can be analyzed in more detail by considering mechanisms by which impacts along one axis reverberate along another axis. The concept of intersectionality was originally developed for understanding how different personal identities or groups to which a person could belong could yield impacts in terms of marginalization that are either magnified or substantially different at intersections of those identities. A classic example is that of marginalization as a member of the group of women, and marginalization as a member of a visible minority, where women who are visible minorities experience marginalization in both of those identities, such that their experiences differ from that of men in the same visible minority, or women who are not a visible minority. This concept of intersection can be applied to these three axes as well, an extension from its original application to identities that highlights a very similar pattern where the axes of impact intersect [50].

Consider the intersection of the Environmental and Health axes. There are mechanisms by which cumulative harm to the environment also results in cumulative harm to local people living in that environment, but does so in ways that cannot be tied to a single project at a time. The BRFN have responded to the environmental assessment for the Site C Dam by conducting their own Traditional Land Use Study (TLUS) pertaining to the Site C Dam [25] (p. 2). According to BRFN members, cumulative industrial effects include, but are not limited to, the following changes:

- Less water, dried up springs and creeks (waterway impacts of forestry and other landscape changes, surface and groundwater extraction by the oil and gas industry);
- Contaminants in water, air, animals, and plants (from source including but not limited to: oil and gas well sites, flaring, spraying, hydroelectric dams, and agricultural runoff); and
- Access roads, seismic lines, well sites, and other land disturbances [28] (p. 28).

Flooding would increase the release of Methyl Mercury into the water which would likely biomagnify and bioaccumulate in fish and soils [51].

Pollutants such as Methyl Mercury accumulate in watersheds such that individual projects may fall below a specified limit for safe release, while the totality of projects far exceed it. The impact such pollutants have can be direct on individuals, if a local water source becomes contaminated; but can also be indirect, when, for instance, fish or other game animals are consumed that have biomagnified the toxins and thus expose people to dramatically elevated levels. The exposure through animal exposure is an example of an indirect harm on health that is mediated by the direct harm on the environment, and is distinct from the direct harm such pollutants may also have on local people. At this intersection of environment and health axes, the negative effects on environment amplify the negative effects of the pollutants on people.

This amplification is heightened when we consider social justice impacts. There are a number of key factors that together result in the abrogation of full Treaty rights for the BRFN bands; the impact of cumulative environmental fragmentation, pollution, and watershed changes impedes the meaningful exercise of treaty rights. It is widely recognized that when a right cannot be meaningfully exercised, it is effectively being violated. The environmental harm is direct when pollutants are released into the watershed. There is indirect amplification of harm on the intersecting health axis, on the health of the local people who may consume animals that have been exposed. There is also further indirect amplification of harm on the intersecting social justice axis through the impedance of full exercise of traditional treaty rights. The right to hunt game and fish freely on these lands is effectively abrogated when the game and fish cannot be safely consumed or even processed in clean waters in traditional ways. Capping pollutant release to certain threshold levels for indicators measuring environmental impacts fails to measure these further impacts on health and social justice.

The way in which environmental harm intersects with social justice harm thus includes the inability to freely exercise treaty rights such as hunting and fishing. But this intersection also has further consequences on health, beyond those just noted. If fish and game are not available because of pollutants in the watershed, those food sources must be replaced with some other food source. This can add both less healthy food items, and additional economic burdens because of the displaced traditional food sources.

A further factor to consider in social justice impacts is the economic boom and bust cycles associated with the massive IRE projects, many of which bring in large numbers of outside workers for comparatively short periods of time. Large IRE projects set up the conditions for sharp booms at the beginning of such projects, with the scarcities that can come with abrupt competition between sometimes thousands of other workers for commodities and housing. Similarly, sharp busts also occur, with the rapid drawing-down of work force levels and a sudden drop in consumption in the area. Boom and bust cycles preclude the kind of stable normal economic development that the bands might otherwise be able to rely on. As such, their economic autonomy is compromised by the outside pressures of these projects and their workforces in a way that is outside of local control or influence. The biological, physiological, mental, and emotional impacts that are felt by community members as well as those employed by the sector can be multifold but are customarily addressed as discrete problems. The biomedical model promotes the idea of an atomized body which medical specialism have been developed to treat illness and disease. This framework is informed by philosophical ideas such as the Cartesian separation of mind from body. As a result, an illness phenomenon like stress tends to be diagnosed and treated as a health issue unrelated to physical ailments. Not only are different dimensions of the body and health separated from one another, individual cases tend to be disaggregated from collective ones. As a result, for problems to be defined as public health issues, individual issues have to be recorded and their accumulation formally tallied for them to be deemed a cluster of impacts which convey a population-wide effect. In addition, in underfunded and under resourced health care systems, like in Northern BC, the capacity of practitioners to focus on upstream prevention is significantly curtailed, particularly when it is already overwhelmed with the challenge of treating individuals presenting with acute health care challenges. The cumulative character of these multifold health impacts can constitute a systemic social justice issue at this intersection of the health and social justice axes.

Finally, consider the very way in which the Blueberry River First Nations bands are consulted for EAs. This is part of the process by which their rights are to be respected in the approval process. This is direct, in that consultation is required by the treaty for such projects. It is also indirect, in that such consultation is the primary vehicle by which the bands may voice concerns over various aspects of a given proposal, such as its potential impact on the watershed or on a particular animal species. However, the sheer volume of such projects also needs to be considered. Each of these projects must involve a consultation with the relevant First Nations bands, but when the scale of the number of projects grows, the consultation process itself becomes an enormous burden on the bands themselves. They are faced with a dubious dilemma. On one horn of the dilemma, they must be involved in a large number of these consultations involving at least some knowledge of each project. This is more than a full time job, even though it is not compensated for in the EA process. The burden of consultation on a project, considered individually, is not particularly onerous. However, the cumulative burden of ongoing consultations for such a large number of projects becomes manifestly burdensome. On the other horn of the dilemma, though, this consultation process is the only avenue within the EA process by which the BRFN may assert control over their territory and by which they can ensure at least some respect for the area. If they do not engage in these consultations in a meaningful way, then problematic projects may get approved that further degrade the meaningful exercise of their treaty rights. Engagement fatigue results from the use of consultation as the primary means of navigating the intersection of these three axes. It places the burden of navigating the IRE with treaty rights disproportionately on the bands themselves. The consultation process itself is required for each project considered individually, thus preventing harm along the social justice axis. Yet considered cumulatively, the sheer number of such consultations can have a different valence as negatively impacting the BRFN along that very same axis.

## 5. Conclusions

As noted above, the term “health” has a double meaning in this context, since it applies both to the health of the people in the area as well as to the health of the ecosystems. Each of these kinds of health can be measured separately along its own axis, and when considered separately, each can also be measured using individual indicators on a project by project basis, or in terms of cumulative impacts. There are further mechanisms by which these two axes interact and amplify harm when they intersect, however, so that both the direct and the indirect implications must be considered to get a fuller view.

This has a variety of implications for assessing projects for approval using the current procedures in place in BC. The BC government has begun moving to a cumulative effect model for understanding environmental impacts; however, the capacity to use a cumulative impacts framework to understand the health impacts produced at the intersection between ecological and social factors—such as treaty rights—is still in its nascent stages. Furthermore, there will need to be movement to a cumulative impacts framework that is also accompanied by an appropriate intersectional analysis of the three axes identified here—environment, health, and social justice—such that the direct and indirect mechanisms of harm can be identified and mitigated without inducing further burdens of consultation and ensuring engagement fatigue.
